# National survey of risk factors for non-communicable disease in Vietnam: prevalence estimates and an assessment of their validity

**DOI:** 10.1186/s12889-016-3160-4

**Published:** 2016-06-10

**Authors:** Tan Van Bui, Christopher Leigh Blizzard, Khue Ngoc Luong, Ngoc Le Van Truong, Bao Quoc Tran, Petr Otahal, Seana Gall, Mark R. Nelson, Thuy Bich Au, Son Thai Ha, Hai Ngoc Phung, Mai Hoang Tran, Michele Callisaya, Velandai Srikanth

**Affiliations:** Menzies Institute for Medical Research, University of Tasmania, Private Bag 23, Hobart, TAS 7000 Australia; Can Tho University of Medicine and Pharmacy, Can Tho, Vietnam; Medical Services Administration, Ministry of Health of the Socialist Republic of Vietnam, Ha Noi, Vietnam; Department of Medicine, Southern Clinical School, Monash Medical Centre, Monash University, Clayton, VIC Australia

**Keywords:** Non-communicable disease, Risk factors, Prevalence, Ecological inference

## Abstract

**Background:**

To estimate the prevalence of non-communicable disease (NCD) risk factors at a provincial level in Vietnam, and to assess whether the summary estimates allow reliable inferences to be drawn regarding regional differences in risk factors and associations between them.

**Methods:**

Participants (*n* = 14706, 53.5 % females) aged 25–64 years were selected by multi-stage stratified cluster sampling from eight provinces each representing one of the eight geographical regions of Vietnam. Measurements were made using the World Health Organization STEPS protocols. Data were analysed using complex survey methods.

**Results:**

Differences by sex in mean years of schooling (males 8.26 ± 0.20, females 7.00 ± 0.18), proportions of current smokers (males 57.70 %, females 1.73 %), and binge-drinkers (males 25.11 %, females 0.63 %), and regional differences in diet, reflected the geographical and socio-cultural characteristics of the country. Provinces with a higher proportion of urban population had greater mean levels of BMI (*r* = 0.82), and lesser proportions of active people (*r* = −0.89). The associations between the summary estimates were generally plausible (e.g. physical activity and BMI, *r* = −0.80) but overstated, and with some anomalous findings due to characterisation of smoking and hypertension by STEPS protocols.

**Conclusions:**

This report provides an extensive description of the sex-specific and regional distribution of NCD risk factors in Vietnam and an account of some health-related consequences of industrialisation in its early stages. The STEPS protocols can be utilized to provide aggregate data for valid between-population comparisons, but with important caveats identified.

## Background

Non-communicable diseases (NCDs) are a leading cause of death worldwide [1]. In Vietnam, there has been a 30 % increase in NCD morbidity and mortality between 1976 and 2009 [2]. This increase may be due, in part, to improved reporting, but ageing of the population and increased exposure to NCD risk factors in a country undergoing rapid urbanisation/industrialization is also likely to be a contributing factor. The NCD risk factors include tobacco smoking, harmful use of alcohol, more sedentary forms of work and leisure, and consumption of energy dense food [1].

Information on the prevalence of NCD risk factors in Vietnam is limited to the urban and affluent cities of Ha Noi [3, 4], Ho Chi Minh City (HCMC) [5–7], and Can Tho [8–12]. Although previous studies [13, 14] collected information across Vietnam, regional comparisons were not presented, different sampling strategies were used, and data for those analyses were collected at various time points (2001–2009). In addition, populations in different ecological regions are likely to have different risk profiles due to variation in numerous socio-demographic factors and lifestyle or pathophysiological factors such as overweight/obesity [15]. Furthermore, about 70 % of the Vietnamese people live in rural areas [16], with information on risk factors unavailable for this sector of the population.

The first aim of this study was to provide summary estimates of the prevalence of NCD risk factors at provincial and national levels. These findings will guide the development of public health policy for NCDs in Vietnam. Because the summary estimates are likely to be used to compare risk factor levels between provinces and to derive inferences about relationships between provincial levels of risk factors, our second aim was to investigate the validity of the summary estimates when used for these purposes. The findings have bearing on the use and value of data collections such as the WHO Global InfoBase, the data warehouse of information on chronic diseases and risk factors for WHO member states. One purpose of the InfoBase is to allow users to compare levels of risk factors across countries. The WHO STEPS methodology [17] is specifically designed to provide summary data that are reliable for cross-cultural comparisons [18–20], but the validity of the summary measurements for this purpose has not been subjected to rigorous examination.

## Methods

### Study participants and sampling

**Fig. 1:**
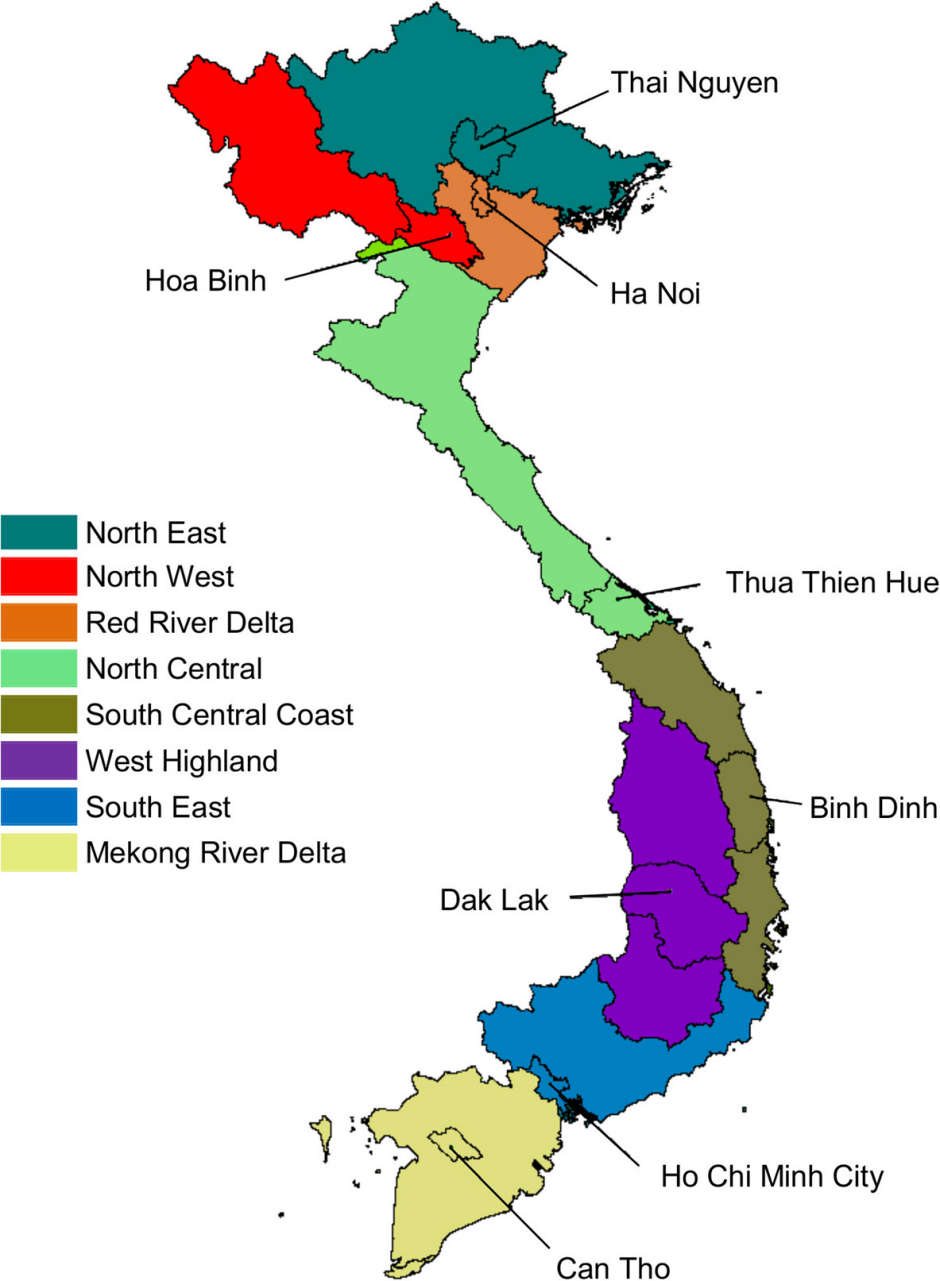
Eight provinces each represent one of the eight ecological regions of Vietnam

This population-based survey was conducted among 25 to 64-year-old residents of eight provinces in Vietnam during 2009/10. The provinces were Thai Nguyen, Hoa Binh, Ha Noi, Thua Thien Hue (Hue), Binh Dinh, Dak Lak, HCMC, and Can Tho (Fig. 1). Each represents one of the eight ecological and geographical regions of Vietnam. Eligible subjects were selected by stratified two-stage cluster sampling. Of the 22,940 eligible subjects selected for participation, 14,706 (64.1 %) participated in this survey. Details of this survey have been reported elsewhere [21].

### Measurements

Socio-demographic information on residential status (urban and rural), ethnicity (the Kinh majority group, and non-Kinh minority groups including Khmer, Tay, Ede, and Chinese), monthly household income per adult household member, years spent at school, and four behavioural factors (tobacco smoking, alcohol, fruit/vegetable consumption, and physical activity) were collected using the STEPS questionnaire [17]. Pathophysiological measurements including weight, height, waist circumference, hip circumference, systolic blood pressure (SBP), diastolic blood pressure (DBP), fasting blood glucose, and fasting total cholesterol were made using the standardised procedures of the STEPS protocols [17]. Data collectors were trained and co-supervised by the Menzies Institute for Medical Research, Tasmania, Australia. The questionnaire was adapted for local use and translated and back-translated. Pilot studies were conducted to test survey instruments and procedures.

### Data analysis

Data were entered and coded in accordance with STEPS protocols [17]. Provincial and national means and proportions were calculated using complex survey methods with sampling weights calculated in accord with the sampling design. Principal component analysis was used to guide the selection of the most comprehensive measure of each risk factor from all measures of it specified by STEPS protocols. An indicator that loaded most heavily upon first principal component, and produced the greatest correlation with relevant and more proximal variables, was selected. Those selected were the proportion of current smokers, binge drinkers (males ≥ five standard drinks, females ≥ four standard drinks in any day last week), respondents with at least 3000 metabolic equivalent of task (MET) – weighted minutes of physical activity per week, raised blood pressure (SBP ≥140 mmHg and/or SBP ≥90 mmHg), raised blood glucose (blood glucose values >6.1 mmol/L or taking medications for diabetes) [17], mean body mass index (BMI, kg/m^2^), number of servings of fruit and vegetables per day, and total cholesterol (mmol/L). Non-missing data were re-weighted to account for missing data [22], and Box-Cox transformations were applied to continuous data (e.g. right-skewed physical activity data). Because a constant needed to be added to data with zero values, and the choice of its values is arbitrary, the constant was selected to make the summary estimate for this design (the mean of cluster means) as close as possible to the median for this design (the median of cluster medians). Pearson correlation coefficients were used to summarise the associations between survey-weighted provincial means of the socio-demographic, behavioural and pathophysiological factors stratified by sex.

## Results

Summary information on the response proportions is presented in Table 1. The overall response proportion was 64.1 % (14,706/22,940). The response proportions generally increased with age, were higher for women than men, and lowest in the two largest cities of Ha Noi and HCMC.

**Table 1:** Response proportions in the national survey of risk factors for NCDs in Vietnam, by age groups and provinces

	Men		Women		Total	
	%	(n/N)	%	(n/N)	%	(n/N)
Age groups						
25–34 years	42.0	(1423/3388)	57.6	(1745/3030)	49.4	(3168/6418)
35–44 years	59.1	(1666/2819)	73.1	(1925/2632)	65.9	(3591/5450)
45–54 years	63.4	(1791/2823)	83.8	(2146/2561)	73.1	(3937/5384)
55–64 years	66.6	(1924/2887)	74.5	(2086/2801)	70.5	(4010/5688)
Provinces						
Thai Nguyen	77.6	(963/1241)	91.6	(1087/1187)	84.4	(2050/2428)
Hoa Binh	66.4	(887/1335)	81.3	(1015/1248)	73.6	(1902/2583)
Ha Noi	45.4	(737/1624)	59.3	(906/1528)	52.1	(1643/3152)
Hue	60.4	(853/1412)	83.0	(1013/1220)	70.9	(1866/2632)
Binh Dinh	70.5	(885/1256)	90.0	(1026/1140)	79.8	(1911/2395)
Dak Lak	55.3	(872/1578)	63.0	(937/1487)	59.0	(1809/3064)
HCMC	40.1	(840/2095)	49.4	(971/1967)	44.6	(1811/4063)
Can Tho	55.8	(767/1375)	75.9	(947/1248)	65.3	(1714/2623)
Total	57.1	(6804/11916)	71.7	(7902/11024)	64.1	(14706/22940)

The summary estimates of socio-demographic, behavioural and pathophysiological characteristics are presented in Table 2. The proportions of urban population were highest in the provinces of HCMC, Can Tho, and Ha Noi where the proportions of physically active people were lowest, and mean BMI was highest. As expected, the proportions of non-Kinh ethnicity were highest in Hoa Binh, Dak Lak, and Thai Nguyen. Mean years of schooling and monthly income were highest in the two largest cities (Ha Noi and HCMC). The proportions of current smokers where highest in the male populations of the central provinces of Binh Dinh and Hue, and high also in Can Tho, where the proportions of binge drinkers also tended to be high. Mean fruit and vegetable consumption was generally highest in the northern provinces of Thai Nguyen, Hoa Binh and Ha Noi. The proportions with elevated glucose were generally higher in the southern-most provinces (Dak Lak, HCMC, and Can Tho), and mean cholesterol were markedly higher in HCMC and Can Tho. The proportions with raised blood pressure were low in the three principal cities (Ha Noi, HCMC, and Can Tho), and high in the mountainous province of Dak Lak. As shown in Table 2, the most notable sex differences were greater mean years of schooling and levels of physical activity and higher prevalence of tobacco smoking, binge drinking and raised blood pressure among men. Despite their lower physical activity, the mean BMI of women was similar to that of men.

**Table 2:** Characteristics of participants from the 8 representative provinces of Vietnam, by sex (*n* = 14706)

	Thai Nguyen		Hoa Binh		Ha Noi		Hue		Binh Dinh		Dak Lak		HCMC		Can Tho		Total	
	Mean	95 % CI	Mean	95 % CI	Mean	95 % CI	Mean	95 % CI	Mean	95 % CI	Mean	95 % CI	Mean	95 % CI	Mean	95 % CI	Mean	95 % CI
Men																		
Urban population^a^ (%)	22.2	±0.00	11.9	±0.00	42.9	±0.00	33.6	±0.00	26.0	±0.00	21.6	±0.00	83.1	±0.00	66.3	±0.00	29.8	±0.00
Minority ethnicity^b^ (%)	14.4	±5.70	74.8	±3.20	0.29	±0.34	0.03	±0.07	0.10	±0.20	20.4	±4.86	5.20	±3.70	1.62	±1.24	5.84	±0.87
Years at school^c^	8.69	±0.31	7.56	±0.26	10.2	±0.51	8.00	±0.46	8.10	±0.33	8.34	±0.48	10.0	±0.45	8.00	±0.65	8.26	±0.20
Household income^d^	52.6	±4.26	21.6	±4.78	89.2	±10.9	41.6	±3.32	50.1	±2.96	45.6	±7.03	91.0	±10.9	44.7	±4.40	53.2	±2.22
Current smoker^e^ (%)	55.5	±4.23	54.4	±11.8	52.5	±4.12	63.4	±3.85	64.8	±3.37	45.0	±5.82	54.2	±4.60	58.3	±5.46	57.7	±1.83
Alcohol intake^f^	17.3	±4.35	20.5	±8.25	16.6	±3.24	34.0	±4.28	28.1	±3.69	29.4	±4.64	22.3	±4.83	30.2	±4.75	25.1	±1.68
Fruit/vegetable serves^g^	3.48	±0.21	2.80	±0.32	3.41	±0.18	2.46	±0.18	2.46	±0.11	2.27	±0.18	2.58	±0.21	2.45	±0.20	2.74	±0.08
Physical activity^h^ (%)	87.9	±3.22	83.5	±7.02	38.4	±5.97	47.3	±4.32	73.2	±2.32	80.0	±3.13	28.7	±4.63	37.2	±4.71	52.0	±2.26
BMI^i^	20.4	±0.21	20.7	±0.46	21.8	±0.29	20.4	±0.21	20.6	±0.20	21.0	±0.23	22.1	±0.30	21.2	±0.33	21.1	±0.11
Raised BP^j^ (%)	20.1	±3.33	20.2	±2.74	15.7	±3.01	16.2	±3.09	21.5	±3.27	25.5	±3.77	17.3	±3.10	17.0	±3.11	18.5	±1.29
Elevated glucose^k^ (%)	1.75	±0.97	2.73	±1.87	2.92	±2.28	1.50	±0.76	1.41	±0.88	3.33	±2.05	3.15	±1.42	3.89	±1.78	2.63	±0.72
Cholesterol^l^	4.26	±0.04	4.46	±0.07	4.64	±0.06	4.47	±0.06	4.52	±0.05	4.51	±0.09	4.73	±0.07	4.80	±0.09	4.58	±0.03
Women																		
Urban population^a^ (%)	22.7	±0.00	12.7	±0.00	43.7	±0.00	34.3	±0.00	26.1	±0.00	22.0	±0.00	84.1	±0.00	67.7	±0.00	30.8	±0.00
Minority ethnicity^b^ (%)	12.3	±4.39	74.7	±6.65	0.94	±0.88	0.10	±0.14	0.26	±0.35	18.2	±5.55	3.59	±2.61	2.80	±1.80	5.41	±0.74
Years at school^c^	8.17	±0.34	7.00	±0.33	9.50	±0.41	5.50	±0.43	6.42	±0.20	7.00	±0.56	9.00	±0.47	6.00	±0.54	7.00	±0.18
Household income^d^	51.5	±4.70	20.4	±3.72	80.2	±6.59	40.0	±2.73	46.4	±2.79	45.0	±6.05	96.0	±10.4	45.5	±3.18	52.9	±2.42
Current smoker^e^ (%)	1.09	±0.86	8.04	±5.96	0.56	±0.45	4.90	±1.34	0.60	±0.53	1.37	±1.01	2.12	±0.93	1.12	±0.60	1.73	±0.32
Alcohol intake^f^	1.22	±0.89	1.48	±2.09	0.35	±0.51	0.44	±0.39	0.34	±0.41	0.10	±0.10	1.38	±0.84	0.88	±1.21	0.63	±0.24
Fruit/vegetable serves^g^	3.18	±0.14	3.37	±0.57	3.58	±0.20	2.65	±0.18	2.34	±0.09	2.27	±0.22	3.05	±0.17	2.36	±0.15	2.80	±0.07
Physical activity^h^ (%)	83.1	±4.12	78.0	±6.10	35.6	±4.36	39.6	±3.88	63.8	±3.59	70.3	±5.39	13.2	±2.32	25.6	±3.65	41.1	±1.48
BMI^i^	20.5	±0.27	20.2	±0.12	21.6	±0.24	20.9	±0.23	20.8	±0.21	20.6	±0.31	21.5	±0.23	21.9	±0.24	21.2	±0.10
Raised BP^j^ (%)	9.05	±2.43	11.8	±3.28	7.69	±1.66	9.93	±1.88	10.4	±1.91	15.7	±3.64	9.09	±1.82	13.3	±2.19	10.2	±0.85
Elevated glucose^k^ (%)	0.84	±0.52	3.48	±3.47	1.84	±0.90	1.95	±0.84	2.58	±1.01	3.10	±1.47	3.28	±1.27	4.55	±1.54	2.58	±0.47
Cholesterol^l^	4.17	±0.04	4.39	±0.05	4.61	±0.07	4.62	±0.06	4.76	±0.05	4.54	±0.07	4.85	±0.06	4.90	±0.08	4.66	±0.03

^a^Proportion of urban population; ^b^Proportion of non-Vietnamese minority ethnic group; ^c^Mean years of schooling; ^d^Mean household income per adult person per month (USD), ^e^Proportion of current smokers; ^f^Proportion with binge drinking (≥4 standard drinks for females, and ≥5 standard drinks for males, on any day last week); ^g^Mean daily servings of fruit and vegetables; ^h^Proportion with high levels of physical activity (≥3000 MET-minutes per week); ^i^Mean BMI (kg/m^2^); ^j^Proportion with raised BP (systolic pressure ≥140 mmHg and/or diastolic pressure ≥90 mmHg); ^k^Proportion with fasting blood glucose values >6.1 mmol/L or taking medications for diabetes; ^l^Mean fasting total cholesterol values (mmol/L)

Correlations between the summary values are shown in Table 3. The urban population proportions co-varied inversely with the provincial proportions of active people (*r* = −0.89, men and women combined) and positively with provincial mean BMI (*r* = 0.82, men and women combined), provincial mean cholesterol and the provincial proportions with elevated glucose. There were generally weaker associations of physical activity, BMI, cholesterol (men) and elevated glucose (men) with provincial mean years of schooling and mean household income, each of which co-varied positively with the urban proportions. In addition, years of schooling and household income were inversely related to the proportions of current smokers and binge drinkers (men). The provincial proportions of minority ethnicity were positively correlated with proportions of active people and negatively with mean levels of BMI, and positively with fruit/vegetable intake (women). Provincial mean BMI was inversely correlated with the proportions of persons reporting high physical activity (*r* = −0.80, men and women combined) and positively correlated with proportions with elevated glucose and mean cholesterol, which co-varied positively (Table 3).

**Table 3:** Correlation coefficients between provincial levels of demographic, behavioural and pathophysiological factors, by sex

Women	Urban pop^a^	Minority ethnicity^b^	Years at school ^c^	H’hold income^d^	Current smokers^e^	Alcohol intake ^f^	Fruit/veg serves^g^	Physical activity^h^	BMI^i^	Raised BP^j^	Elevated glucose^k^	Cholesterol^l^
Men												
Urban population^a^ (%)		−0.5^m^	0.27	0.73	−0.34	0.23	−0.04	−0.92	0.86	−0.20	0.41	0.75
Minority ethnicity^b^ (%)	−0.52		−0.06	−0.58	0.79^m^	0.50^m^	0.35	0.55	−0.62	0.28	0.24	−0.49
Years at school^c^	0.56^m^	−0.44		0.76	−0.31	0.25	0.72	−0.16	0.21	−0.55	−0.33	−0.16
Household income^d^	0.65	−0.59^m^	0.98		−0.56^m^	0.03	0.30	−0.66	0.62	−0.53^m^	−0.14	0.39
Current smoker^e^ (%)	0.05	−0.32	−0.32	−0.18		0.45	0.29	0.23	−0.50^m^	0.09	0.18	−0.29
Alcohol intake^f^	0.07	−0.29	−0.55^m^	−0.41	0.36		0.49	0.00	−0.10	−0.24	0.15	−0.22
Fruit/vegetable serves^g^	−0.16	0.06	0.46	0.34	−0.13	−0.87^m^		0.03	−0.06	−0.68	−0.40	−0.45
Physical activity^h^ (%)	−0.88	0.57	−0.56	−0.64	−0.16^n^	−0.14	0.12		−0.89	0.25^n^	−0.38	−0.82
BMI^i^	0.78	−0.25	0.82	0.82	−0.39	−0.32	0.07	−0.74		−0.18^n^	0.38	0.77
Raised BP^j^ (%)	−0.57^m^	0.35	−0.39	−0.42	−0.43	0.13	−0.33	0.77^n^	−0.39^n^		0.61	0.10
Elevated glucose^k^ (%)	0.50^m^	0.12	0.22	0.19	−0.63^m^	−0.05	−0.19	−0.39	0.65	−0.02		0.65
Cholesterol^l^	0.82	−0.33	0.33	0.43	−0.03	0.22	−0.39	−0.82	0.76	−0.41^n^	0.71	

^a^Proportion of urban population; ^b^Proportion of non–Vietnamese minority ethnic group; ^c^Mean years of schooling;^d^Mean household income per adult person per month (USD), ^e^Proportion of current smokers; ^f^Proportion with binge drinking (≥4 standard drinks for females, and ≥5 standard drinks for males, on any day last week); ^g^Mean daily servings of fruit and vegetables; ^h^Proportion with high levels of physical activity (≥3000 MET–minutes per week); ^i^Mean body mass index (kg/m^2^); ^j^Proportion with raised blood pressure (systolic pressure ≥140 mmHg and/or diastolic pressure ≥90 mmHg); ^k^Proportion with fasting blood glucose values >6.1 mmol/L or taking medications for diabetes; ^l^Mean fasting total cholesterol values (mmol/L); ^m^Weaker individual–level correlation [absolute(r_province_) ≥0.5 but absolute(r_individual_) <0.05]; ^n^ Individual–level correlation of opposite sign (r_province_ >0.10 and r_individual_ > −0.10 or r_province_ > −0.10 and r_individual_ >0.10)

The provincial-level associations were generally larger than the corresponding strength of that association in individual-level data. Some that were disproportionately larger have been highlighted in Table 3 (see ‘m’ symbol). Several of these involved either the proportions of current smokers or those with raised blood pressure. In addition, there were some associations of opposite sign in individual-level data (see ‘n’ symbol, Table 3). Most of these were associations with raised blood pressure. As examples, the proportions with raised blood pressure were positively associated with proportions of active persons and negatively associated with mean levels of BMI (and with mean waist circumference and waist-to-hip ratios, data not shown).

**Fig. 2:**
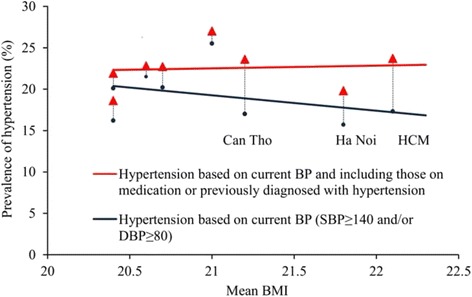
Associations between provincial proportions of men with hypertension and provincial mean BMI of those men

This placed suspicion on its definition in STEPS protocols as SBP ≥140 mmHg and/or DBP ≥90 mmHg. This definition does not account for blood pressure treated by antihypertensive medication or other means. As shown in Table 4, the proportions of people taking prescribed medication for raised blood pressure, and the proportions of respondents who reported having been previously diagnosed with hypertension, were markedly higher in Can Tho and HCMC, and among men from Ha Noi. Including those on prescribed medication or previously diagnosed with hypertension in the definition of raised blood pressure increased the estimated national proportion with raised blood pressure by around four percentage points with the largest increases in Can Tho, HCMC and Ha Noi (men). Doing so also substantially reduced its uncharacteristic positive association of the provincial proportions with physical activity for men, and reversed each of the other anomalous associations. A demonstration of how this occurred for mean BMI is presented in Fig. 2.

**Table 4:** Participants who reported taking prescribed medication for raised blood pressure (BP) or having been diagnosed previously with hypertension, and estimated prevalence of hypertension with these factors taken into account

	Thai Nguyen		Hoa Binh		Ha Noi		Hue		Binh Dinh		Dak Lak		HCMC		Can Tho		Total	
	Mean	95 % CI	Mean	95 % CI	Mean	95 % CI	Mean	95 % CI	Mean	95 % CI	Mean	95 % CI	Mean	95 % CI	Mean	95 % CI	Mean	95 % CI
Men																		
BP medication^a^	0.82	±0.64	1.57	±1.71	1.71	±0.61	1.56	±0.59	1.32	±0.56	1.89	±1.25	3.98	±1.28	4.23	±1.62	2.35	±0.49
Prior diagnosis^b^	6.70	±1.75	6.32	±2.10	7.96	±2.14	5.55	±1.41	5.59	±1.35	6.23	±1.98	10.1	±1.96	12.4	±2.79	8.21	±0.88
Raised BP 1^c^	20.1	±3.33	20.2	±2.74	15.7	±3.01	16.2	±3.09	21.5	±3.27	25.5	±3.77	17.3	±3.10	17.0	±3.11	18.5	±1.29
Raised BP 2^d^	20.3	±3.31	21.3	±3.62	16.2	±3.03	16.9	±3.06	21.6	±3.28	25.8	±3.77	19.3	±3.19	18.8	±3.35	19.4	±1.38
Raised BP 3^e^	21.9	±3.12	22.7	±3.75	19.8	±3.50	18.6	±3.16	22.8	±3.22	27.0	±3.72	23.7	±3.36	23.6	±3.42	22.4	±1.51
Women																		
BP medication^a^	1.29	±0.52	1.50	±1.02	2.52	±0.94	3.48	±1.01	2.97	±0.82	2.07	±1.31	4.49	±1.26	7.95	±1.95	3.55	±0.47
Prior diagnosis^b^	5.69	±1.63	5.54	±4.51	6.82	±1.56	8.18	±1.63	7.92	±1.40	8.66	±2.85	8.53	±1.86	15.9	±2.23	8.75	±0.81
Raised BP 1^c^	9.05	±2.43	11.8	±3.28	7.69	±1.66	9.93	±1.88	10.4	±1.91	15.7	±3.64	9.09	±1.82	13.3	±2.19	10.2	±0.85
Raised BP 2^d^	9.64	±2.47	12.0	±3.27	8.51	±1.72	10.8	±1.90	11.1	±1.80	16.0	±3.64	11.1	±2.07	16.9	±2.37	11.5	±0.90
Raised BP 3^e^	11.2	±2.52	15.2	±4.58	10.9	±1.98	13.2	±2.08	13.0	±1.77	18.9	±3.66	14.2	±2.30	22.7	±2.55	14.6	±1.01

^a^Taking medication for raised blood pressure, as indicated by a positive responses to the question “During the past two weeks, have you been treated for raised blood pressure with drugs (medication) prescribed by a doctor or other health worker?”

^b^Previously diagnosed with hypertension, as indicated by a positive response to the question “Have you ever been told by a doctor or other health worker that you have raised blood pressure or hypertension?”

^c^Systolic blood pressure ≥140 mm Hg and/or diastolic blood pressure ≥90 mm Hg

^d^Systolic blood pressure ≥140 mm Hg and/or diastolic blood pressure ≥90 mm Hg or taking medications for hypertension

^e^Systolic blood pressure ≥140 mm Hg and/or diastolic blood pressure ≥90 mm Hg or taking medications for hypertension or previously diagnosed with hypertension

With raised blood pressure defined to include those on prescribed medication or otherwise previously diagnosed with hypertension, the estimated national proportion with raised blood pressure is around four percentage points higher with the largest increases in Can Tho, HCMC and Ha Noi (men).

**Fig. 3:**
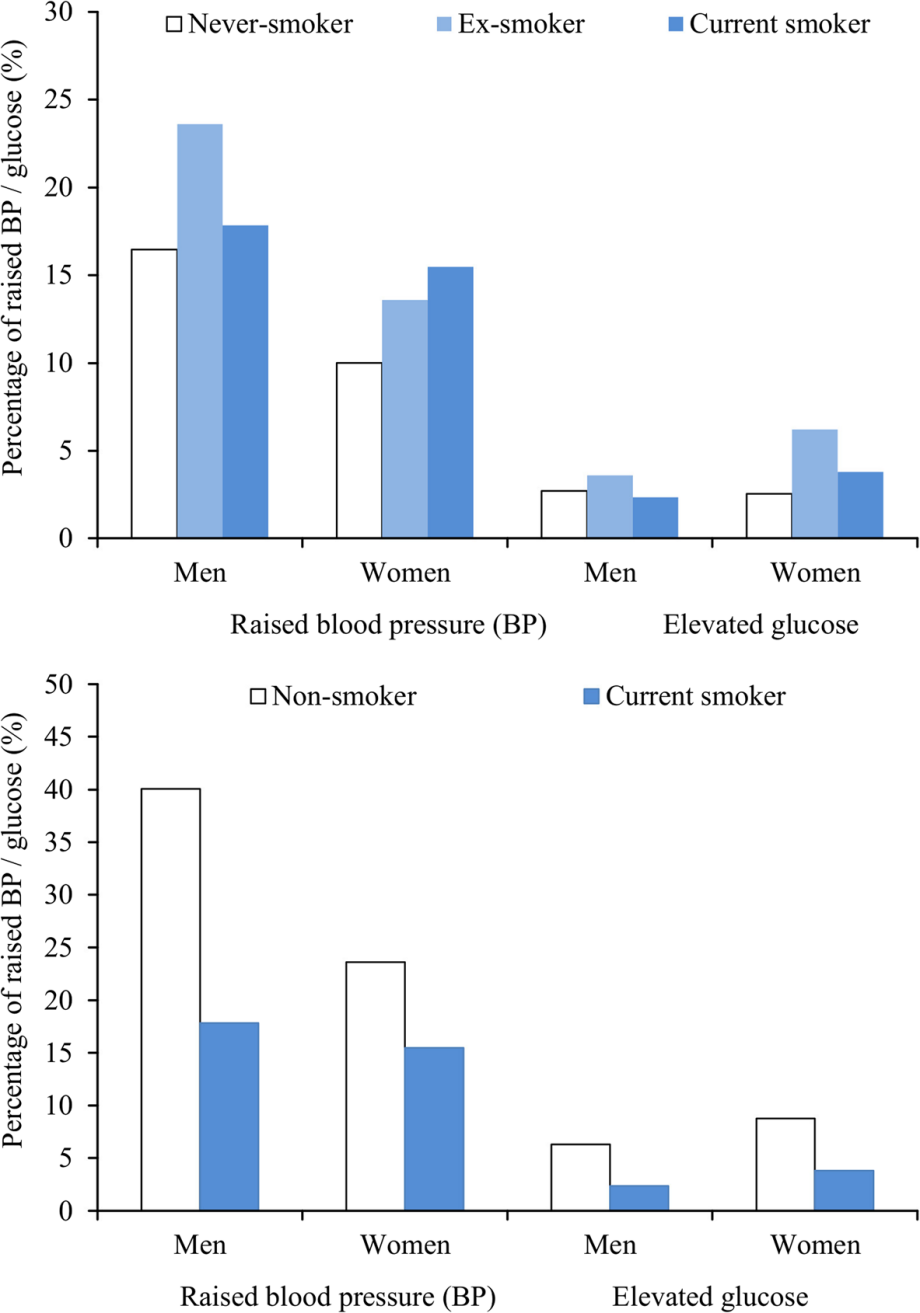
Proportions of respondents with raised blood pressure (BP) and elevated glucose classified by smoking status (Top: never-, ex- and current smoker; Bottom: non- and current smoker)

There were other unexpected associations for current smoking among men. Figure 3 shows that higher proportions of ex-smokers than never-smokers or current smokers had elevated blood pressure and glucose (top panel). Including ex-smokers with never-smokers in the reference category produced the anomalous finding of a negative association between current smoking and hypertension (bottom panel).

## Discussion

This paper presents a comprehensive account of the regional distribution of NCD risk factors in Vietnam made in a standardised way. Participants in the survey were selected using population-based sampling from eight provinces representing eight different geographical regions of Vietnam according to WHO STEPS protocols [17]. Our findings reflect some known regional attributes and social characteristics of the country, and document in aggregate some of the health-related consequences for a developing country in the early stages of economic transition. For the most part, the directions of the associations found in aggregate between provincial levels of the risk factors were as expected from sociological, epidemiological and biological evidence about plausible causal pathways. However, there were unexpected associations with smoking and hypertension. These were due to the characterisation of smoking and hypertension by STEPS protocols.

The information on socio-demographic characteristics mostly accords with official statistical records of the Vietnamese population [16, 23]. The lifestyle characteristics also reflected some of the cultural practices of the country. More than one-half of the men were current smokers and around a quarter of men participated in heavy drinking occasions, whereas those behaviours were rare among women. This has been described previously [3, 8, 24, 25]. Higher proportions of men than women had high levels of physical activity, which is consistent with results of previous studies in Ha Noi [26], HCMC [7], and Can Tho [9]. Non-Kinh subjects living in predominately rural locations were relatively physically active and lean on average. The women among them tended to have higher servings of fruit/vegetables than their Kinh counterparts.

The summary information on health-related behaviours and pathophysiological outcome factors demonstrates the changing NCD risk factor profile of a country undergoing demographic and economic transition. For example, greater schooling and income was associated with reduced smoking, less hazardous/harmful alcohol intake and improved diets on the one hand, and reduced physical activity and higher BMI on the other, in line with previous research on socioeconomic factors and smoking [27], at-risk drinking [25], and improved fruit/vegetable intake [28], physical activity [7, 26], and BMI [6, 15]. The proportions of active people were inversely correlated with the proportion of urban-dwellers. For instance, activity proportions were lowest in Can Tho (two-thirds urban) and HCMC (around 80 % urban). These provinces had the highest mean BMI, highest proportions with elevated glucose and highest mean cholesterol. These correlations between provincial levels of physical activity, BMI, elevated glucose and cholesterol consistent in sign with results at the individual level.

The regional and sex differences in the NCD risk reflect the socio-demographic and cultural characteristics of the country. Smoking and binge drinking were largely confined to men, and to those with lower levels of education. Fruit and vegetable and vegetable intake was higher among persons with higher income. Vegetable consumption was greater in northern provinces, and physical activity was lower in urban areas. These observations attest to the construct validity of the STEPS questionnaire for use in Vietnam. Furthermore, the relationships between risk factors discussed to this point appear sociologically and biologically plausible. Whilst our data are cross-sectional, the associations are consistent with the changing risk factor profile of a developing country undergoing industrialization/urbanisation. Vietnam has experienced increasing urbanisation in recent years [29], with increased adiposity and hypertension a predicted consequence [14, 15]. The process of transition from a traditional/rural to a more modern/urban society is accompanied by a shift from physically active occupations such as farming and forestry toward more sedentary, office-based occupations. For example, national survey data from China during 1991–2006 [30] showed that more than four-fifths of the decline in occupational physical activity for men and nearly two-thirds of the decline for women were predicted by factors associated with urbanisation (e.g. population size and economic well-being). Other research findings have suggested that urbanisation is associated with a higher prevalence of overweight/obesity [31, 32], hypertension [32], and diabetes [31].

The summary estimates for each province were presented in a report prepared for the Ministry of Health of the Socialist Republic of Vietnam, and stored also on a database that could be accessed by staff of the Ministry of Health and of the provincial health authorities. It soon became clear that one of the principal uses of the paper-based and electronic information would be to draw associative inferences (e.g. “the mean level of physical activity in our province is higher than that in other provinces, so is our mean BMI correspondingly lower?”). Aware of this, summary provincial estimates of NCD risk factors and measures of association (correlations) between those summary estimates are presented, together with a warning (by way of footnotes to Table 3) when the provincial-level correlations are not consistent with individual-level associations. The provincial-level associations were generally greater in magnitude than the individual-level associations, however, and in highlighted cases the exaggeration was pronounced. This serves as a first warning about drawing associative inferences from the aggregate data: the provincial-level associations in this study were overstated in the main. A second warning is that in some cases the relationships were not plausible. This was the case for two sets of relationships, and an explanation is provided for each.

The first set of implausible relationships occurred for tobacco smoking. For women, the proportions of smokers (and binge drinkers) were so low that minor differences in proportions have to be discounted due to sampling error. For men, current smoking was inversely related to the proportions with raised blood pressure and glucose because those at highest risk were ex-smokers. Our group identified the hypertension phenomenon previously in a survey in Can Tho [10], and proposed that this was likely due to smokers being prompted to quit by a diagnosis of hypertension. The STEPS protocols allow information to be captured on ex-smokers, but the core instrument refers exclusively to current smokers and the survey report template requires reporting only of the proportion of current daily smokers and their years of smoking and quantities smoked. Our results indicate that information solely on current smokers does not accurately portray the risk profiles of Vietnamese men.

The second anomaly related to raised blood pressure assessed in accordance with STEPS protocols (SBP ≥140 mmHg and/or DBP ≥90 mmHg). While the prevalence of uncontrolled raised blood pressure is an important health system indicator, our results demonstrate that this definition may lead to implausible associations with other risk factors including high physical activity, mean BMI and cholesterol. The implausible associations were resolved by including those using medication for, or previously diagnosed with, hypertension in the definition of raised blood pressure. We would therefore encourage those using STEPS protocols to consider the definition of raised blood pressure that is appropriate for their population, and to be aware that the use of the recommended definition may cause spurious associations. With this expanded definition, our estimates for Vietnam (22.4 % for men and 14.6 % for women) are more similar to those from a previous multi-province study (24.1 % for men and 17.9 % for women aged 25–64 years) [13] that included relatively more participants from urban areas (where hypertension is more prevalent). Our sample accurately reflected the urban-rural population division.

This was the first ecological analysis of the population prevalence of NCD risk factors in Vietnam using a representative sampling frame. To minimise avoidable sources of random error and bias, the measurements were made by trained staff in accordance with standardised protocols designed specifically by WHO for providing data that are culturally-relevant yet valid for international comparisons. The aggregate estimates were shown to have evidence of construct validity and, for the most part, associative validity because relationships between risk factors were of the expected sign. Further, confirmation was provided of the utility of STEPS protocols for the intended purpose of providing aggregate data for valid inter-country comparisons albeit through the prism of intra-country comparisons.

Nevertheless, our study has limitations. First, whilst the response proportion (64 %) was high for a study requiring lengthy clinic attendance with invasive procedures including blood-sampling, it was nevertheless low enough to allow the possibility of non-participation bias. Second, information providing a more thorough understanding of the relationships studied − such as dietary fat, 24-hour urinary sodium, physical activity by objective methods, and ambulatory blood pressure − was not collected. The STEPS method emphasises that small amounts of good quality data are more valuable than large amounts of poor quality data, and focuses on a limited range of data collection made in the best manner possible in large-scale fieldwork. Third, each of the measurements has several alternative forms or quantitative scales, and reporting each is impractical within this limited space. We followed STEPS protocols where possible, and used principal components analysis to select a single indicator per risk factor, and reported more fully in two cases (current smoking and hypertension) when the choice was nuanced or resulted in misinterpretations. This highlighted the importance of the definition of hypertension but, as a fourth limitation, we cannot discount that other factors such as measurement errors (perhaps due to faulty recall or to poor equipment or technique in diagnosis) can account for the sizeable numbers of respondents reporting a previous diagnosis of hypertension in HCMC and Can Tho, despite their blood pressure measurement with automated equipment in accordance with strict protocols in this survey being below the thresholds. A sixth limitation is that data were collected by provincial data collection teams, and inter-team measurement variation cannot be excluded as a contributing factor for part of the differences found between ecological regions. Finally, we tested the validity of the summary estimates for inter-country comparisons through the prism of inter-province comparisons within one country. This is reasonable because the provinces of Vietnam differed widely in terms of socio-demographic factors. Nevertheless, inter-country comparisons could involve considerably more heterogeneity than this.

## Conclusions

In summary, this report provides an extensive description of the sex-specific and regional distribution of NCD risk factors in Vietnam and an account of some health-related consequences of the early stages of urbanisation/industrialization in a developing country. The findings provide information that will be valuable in guiding the development of public health policy in respect of NCDs in Vietnam. In addition, they lend support to the case that STEPS protocols have utility for the intended purpose of providing aggregate data for valid between-population comparisons, but with important caveats identified.

## Abbreviations

BMI, Body Mass Index; BP, Blood pressure; CI, Confidence interval; DBP, Diastolic blood pressure; MET, Metabolic Equivalent Task; NCD, Non-communicable disease; SBP, Systolic blood pressure; STEPS, STEPwise approach to surveillance of non-communicable disease; WHO, World Health Organization
